# Combination of lutetium-177 labelled anti-L1CAM antibody chCE7 with the clinically relevant protein kinase inhibitor MK1775: a novel combination against human ovarian carcinoma

**DOI:** 10.1186/s12885-018-4836-1

**Published:** 2018-09-25

**Authors:** Dennis Lindenblatt, Nastassja Terraneo, Giovanni Pellegrini, Susan Cohrs, Philipp René Spycher, David Vukovic, Martin Béhé, Roger Schibli, Jürgen Grünberg

**Affiliations:** 1 Center for Radiopharmaceutical Sciences ETH-PSI-USZ, Paul Scherrer Institute, 5232 Villigen PSI, Switzerland; 2 Institut for Veterinary Pathology, University of Zurich, Zurich, Switzerland; 3 Department of Chemistry and Applied Biosciences, ETH Zürich, Zurich, Switzerland

**Keywords:** Protein kinase inhibitor, MK1775 (AZD1775), Ovarian carcinoma, L1CAM, mAb chCE7, ^177^Lu-radioimmunotherapy, Combination therapy

## Abstract

**Background:**

Protein kinase inhibitors (PKIs) are currently tested in clinical studies (phase I-III) as an alternative strategy against (recurrent) ovarian cancer. Besides their anti-tumour efficacy, several PKIs have also shown radiosensitizing effects when combined with external beam radiation. Based on these results we asked if the addition of PKIs offers a therapeutic opportunity to improve radioimmunotherapy (RIT) against ovarian cancer. Five PKIs (alisertib, MK1775, MK2206, saracatinib, temsirolimus) were chosen for cytotoxicity screenings based on their current clinical trials in the treatment of ovarian cancer and their influence on cell cycle regulation and DNA damage repair pathways. We combined selected PKIs with ^177^Lu-labelled anti-L1CAM monoclonal antibody chCE7 for our investigations.

**Methods:**

PKIs cytotoxicity was determined via cell colony-forming assays. Biomarker of DNA double-strand breaks (DSBs, γH2A.X) was analysed by western blot and fluorescence microscopy. Flow cytometric measurements were performed to evaluate levels of apoptosis based on mono- or combination treatments. The best combination was used for in vivo combination therapy studies in nude mice with SKOV3ip and IGROV1 human ovarian cancer xenografts. Bonferroni correction was used to determine statistical significance for multiple comparisons.

**Results:**

The highest cytotoxicity against both cell lines was observed for MK1775 and alisertib. Combinations including ^177^Lu-labelled mAb chCE7 and MK1775 decreased ^177^Lu-DOTA-chCE7 IC_60_-values 14-fold, compared to 6-fold, when the radioimmunoconjugate was combined with alisertib. The most effective PKI MK1775 was further evaluated and demonstrated synergistic effects in combination with ^177^Lu-DOTA-chCE7 against IGROV1 cells. Significantly higher amounts of DSBs were detected in IGROV1 cells after combination (91%) compared to either treatment alone (MK1775: 52%; radioimmunoconjugate: 72%; *p* < 0.0125). Early-apoptosis was significantly enhanced in IGROV1 cells correlating with induced DSBs (^177^Lu-DOTA-chCE7: 8%, MK1775: 28%, ^177^Lu-DOTA-chCE7 + MK1775: 40%, *p* < 0.0125). Immunohistochemistry analysis of γH2A.X expression levels after therapy in SKOV3ip xenografts revealed a high sensitivity of the tumour cells to MK1775 and a high radioresistance. A prominent effect of tumour growth inhibition of the RIT and of the combination therapy was observed in vivo in a late stage IGROV1 xenograft model.

**Conclusions:**

Our results warrant further evaluation of combination of MK1775 and radioimmunotherapy.

**Electronic supplementary material:**

The online version of this article (10.1186/s12885-018-4836-1) contains supplementary material, which is available to authorized users.

## Background

Today, epithelial ovarian cancer (OC) represents the most severe gynaecological cancer in women. Approximately 75% of the affected patients are being diagnosed with advanced-stage III or IV, since diagnostic markers and clinical symptoms during early stages are mostly absent [1]. Cytoreductive surgery and paclitaxel-platinum chemotherapy represent the most efficient therapy options for advanced OC. Despite good response rates towards chemotherapies (50–80%), the major issue stays the rather high relapse rate in patients within 2 years including development of taxane or platinum resistant tumours [2, 3].

Modern chemotherapeutic agents like etoposide, gemcitabine, topotecan or pegylated liposomal doxorubicin are frequently used against recurrent ovarian cancers, but unfortunately, with very limited response rates between 15 and 30% [4–7].

Given the high relapse rate of recurrent platinum-resistant OC, overall survival is still limited (approx. 40%) [8]. Alternative screening and therapy strategies are urgently needed. New targeted therapies have been investigated during the last couple of years with the goal to increase overall survival and reduce side-effects. Therefore, various monoclonal antibodies (mAbs) have been studied as a targeted treatment alternative [9]. Unfortunately, mAb-based mono- or combination therapies showed only limited clinical efficacies against advanced OC despite promising pre-clinical results [10–13]. Many research groups focus on alternative therapeutic strategies including several protein kinase inhibitors (PKIs) targeting different signalling pathways or the DNA repair machinery of cancer cells. Besides their efficacy against (recurrent) OC, several PKIs also demonstrated radiosensitizing effects when combined with ionizing radiation (external beam radiation, EBRT) [14–16].

In previous studies we demonstrated the efficacy of anti-L1CAM radionuclide-labelled mAb chCE7 (^177^Lu,^161^Tb, ^67^Cu) as monotreatment or when combined with the chemotherapeutic paclitaxel (PTX) against disseminated OC [17–20]. Based on these findings we explored a new approach investigating the potential synergy between PKIs and anti-L1CAM radioimmunotherapy (RIT) against OC. In this context is worth mentioning that high expression of L1CAM in OC is associated with the rapid growth of aggressive tumours and a poor prognosis for the patients [21]. L1CAM “is a major driver for tumour cell invasion and motility” [22].

Five PKIs were chosen for initial cytotoxicity screenings based on their current clinical studies (OC clinical trials I-II) and their potential radiosensitizing characteristics. Briefly, PKIs candidates were chosen as follows:

Alisertib, an Aurora kinase A PKI, showed modest results in a clinical phase II trial against platinum-resistant or refractory OC [23]. Additionally, a phase I trial is ongoing for the therapy of recurrent OC in combination with the chemotherapeutic PTX (clinical trial identifier: NCT01091428). Alisertib has shown to sensitize glioblastoma cells against radiation in vitro [14].

MK1775 also known as AZD1775 is an ATP-competitive inhibitor of Wee1 tyrosine kinase. Wee1 is one of two kinases (Wee1 and Mik1) that catalyse inhibitory phosphorylation on the CDC2-cyclin B complex [24, 25]. Inhibition of Wee1 abrogates the G2/M arrest and leads to compelled progression to mitosis despite damaged DNA resulting in mitotic catastrophe [26]. MK1775 is under investigation in different clinical trials (Phase II) in combination with gemcitabine, carboplatin or paclitaxel against OC (clinical trial identifier: NCT02101775 and NCT02272790) and has previously shown radiosensitizing characteristics in vivo when combined with ionizing radiation for the treatment of intrinsic potine gliomas [27].

Temsirolismus is a mTOR inhibitor which has shown modest, but not sufficient anti-tumour activity in a recent phase II clinical trial as a monotherapy against persistent/recurrent OC [28, 29]. A clinical phase II study where temsirolimus was combined with bevacizumab has been completed but no study results have been published yet (clinical trial identifier: NCT01010126). The radiosensitizing abilities of temsirolismus were shown in vivo in a glioblastoma model [30].

Saracatinib is an inhibitor of Src tyrosine kinase and was investigated in a phase II trial assessing its use in combination with PTX and carboplatin in advanced OC (clinical trial identifier: NCT00610714). However, results remain to be published. Saracatinib revealed sensitizing effects towards ionizing radiation in a lung cancer in vitro model [16].

MK2206 is a highly selective inhibitor of Akt kinase and was tested in a phase II clinical trial against platinum-resistant OC (clinical trial identifier: NCT01283035) [31]. The study has been recently completed. In addition it was demonstrated in vitro, that MK2206 can sensitize triple negative breast cancer cells towards EBRT [32].

After initial in vitro screenings, we further investigated combinations including PKI MK1775 and the ^177^Lu-labelled mAb chCE7 in vivo. Two OC cell lines (IGROV1, p53wt, SKOV3ip, p53del) were used in our studies.

## Methods

### Cell lines, culture conditions and antibody formats

L1CAM positive SKOV3ip cells were kindly provided by P. Altevogt (German Cancer Research Center, Heidelberg, Germany). Cells were established from ascitic fluid of a nu/nu mouse that was previously injected with SKOV3 cells [33]. SKOV3ip cells were maintained in DMEM medium at 37 °C. IGROV1 cells were a kind gift by Dr. Cristina Müller (Center for Radiopharmaceutical Sciences, Paul Scherrer Institute) and were maintained in RPMI 1640 medium at 37 °C. Both cell media were supplemented with 10% fetal calf serum (FCS), 2 mM glutamine, 100 units/ml penicillin, 100 μg/ml streptomycin and 0.25 μg/ml fungizone (BioConcept, Allschwil, Switzerland). The two cell lines were cultivated in a humidified atmosphere containing 5% CO_2_. Cell lines were authenticated by STR profiling (Department of Molecular Medicine, Aarhus University Hospital, Denmark; DSMZ Authentication Service, Braunschweig, Germany) and were mycoplasma free (Mycoplasma test kit, AppliChem GmbH, Darmstadt, Germany). L1CAM expression in both cell lines was confirmed by flow cytometry (Additional file 1: Figure S1). The cell lines did not require ethics approval.

Chimeric monoclonal antibody mAb chCE7 (human κ light chain and human γ1 heavy chain) is an IgG1-subtype. MAb chCE7 was produced in HEK293 cells and subsequently purified from cell culture supernatant as previously described by Grünberg et al. [34].

### Ligand substitution and ^177^Lu antibody radiolabelling

For ligand substitution, molar excess of p-SCN-Bn-DOTA (Macrocyclics, Dallas, TX, USA) was individually adopted to vary DOTA-to-mAb ratios for in vitro and in vivo experiments, respectively as described elsewhere [20]. Briefly, p-SCN-Bn-DOTA was mixed with mAb chCE7 in 0.1 mol/L sodium phosphate buffer (pH 7.2). The pH was adjusted to pH 9–10 using a saturated Na_3_PO_4_ solution and was incubated over night at 30 °C with gentle shaking. Using a NAP-5 column (GE Healthcare, Glattbrugg, Switzerland) excess ligands were removed and buffer was exchanged into 0.25 M CH_3_COONH_4_ (pH 5.5). Immunoconjugates were concentrated to 5 mg/ml and stored at − 80 °C. The number of chelators coupled to the mAb was determined by mass spectroscopy [17]. For radiolabelling, the radionuclide ^177^Lu (ITG, Garching, Germany) was used 1 to 3 days post-specified calibration date. In brief, 100–450 MBq of ^177^Lu was added to 150 μg immunoconjugate and incubated in 0.25 M CH_3_COONH_4_ buffer (pH 5.5) at 37 °C for 1 h. After radiolabelling, EDTA was added to the reaction mixture (5 min) to a final concentration of 5 mM to complex free ^177^Lu. Radiolabelled antibodies were purified via FPLC size exclusion chromatography on a Superose 12 column (GE Healthcare, Glattbrugg, Switzerland) using phosphate-buffered saline (PBS) as eluent with a flow rate of 0.5 ml/min. Radioimmunoconjugates (RICs) eluted with a retention time of 21 min [19]. The immunoreactive fraction [35] of labelled antibody conjugates ranged from 60 to 83%.

### In vitro cytotoxicity assays

In order to investigate the sensitivity of SKOV3ip and IGROV1 cells towards selected PKIs (alisertib, MK1775, MK2206, saracatinib, temsirolimus; all from Selleckchem, LuBioScience, Luzern, Switzerland) were solved in DMSO and the accordant half-maximal inhibitory concentration (IC_50_) was determined via adapted colony-forming assays [36]. OC cells were seeded into 6-well plates with a density of 250 cells/well and incubated over night at 37 °C. After adhesion cells were washed with PBS and incubated with the relevant PKI with concentrations ranging from 0.1 to 1000 nM for 48 h at 37 °C. Cells were washed with PBS and covered with regular supplemented cell culture medium as previously described. Eleven to fourteen days post plating, colonies were washed with PBS, fixed and stained with crystal violet staining solution containing 0.05% crystal violet, 1% formaldehyde and 1% MeOH for 15 min at room temperature (RT). Colonies were washed twice with PBS and manually counted ([36], adapted). Colonies containing more than 100 cells were considered for counting.

In combination experiments, cells were incubated with the half of the half-maximal inhibitory concentration (half IC_50_) of PKIs for 48 h at 37 °C. 1 ml ^177^Lu-DOTA-chCE7 solution was added either before, simultaneously, or post PKI application in concentrations ranging from 0.05 to 5 MBq/ml for 8 h at 37 °C. The incubation medium was removed at the indicated time points and cells were washed with PBS and incubated with regular supplemented cell culture medium. Again, 11–14 days post plating, colonies were washed with PBS, fixed, stained and counted as described.

### Western blot analysis

Cells were exposed to accordant mono- or combination treatments including IC_50_ of MK1775 (300 nM) for 48 h and/or 5 MBq/ml ^177^Lu-DOTA-chCE7 (5 ml) for 8 h. Cell lysates were subject to SDS-polyacrylamide gelelectrophoresis. Proteins were transferred onto polyvinylidene difluoride (PVDF) membranes (Immobilon, Merck, Schaffhausen, Switzerland) via a semi-dry blotting device (Bio-Rad Laboratories AG, Reinach, Switzerland) and incubated with primary anti-CDC2 (1/1000) -pCDC2 (Tyr-15; 1/1000) or -GADPH (1/1000) antibodies (Cell Signaling Technology, Bioconcept, Allschwill, Switzerland). Detection of proteins was performed with secondary anti-mouse IgG HRP-linked antibody (1/2000, 30 min, RT; Cell Signaling Technology, Bioconcept, Allschwill, Switzerland) and ECL chemiluminescence kit (Perbio Science Switzerland S.A., Lausanne, Switzerland). All antibody dilutions were made in TBST/2% BSA and were incubated over night at 4 °C.

For the detection of histone phosphorylation γH2A.X (Ser-139) Abcam’s histone extraction protocol was used. Briefly, cells were detached from plates and washed twice with ice-cold PBS containing 5 nM sodium butyrate to retain levels of histone acetylation. Washed cells were resuspended and incubated for 10 min in triton extraction buffer (TEB) containing 0.5% triton-X 100 (*v*/v), 2 mM phenylmethylsulfonylfluoride (PMSF) and 0.02% (*w*/*v*) NaN_3_. Suspended cells were centrifuged at 2000 rpm for 10 min at 4 °C, washed in half the volume of TEB buffer, centrifuged again (2000 rpm/10 min/4 °C) and incubated overnight (4 °C) in 0.2 N HCl (75 μL) to extract histones. The next day, cells were again centrifuged as before and the protein concentration of the supernatant determined via advanced protein assay reagent (Cytoskeleton, Inc., Denver, USA). Aliquots were stored at − 20 °C and subjected to western blot analysis as previously described.

### Fluorescence microscopy

Cells were seeded in an 8-well chamber system (Nunc Lab-Tek II Chamberslides, Thermo Fisher Scientific, Reinach, Switzerland) with 15.000 cells per chamber and incubated over night at 37 °C. After adhesion cells were treated with MK1775 (100 nM, 300 μl) for 48 h and/or 2.5 MBq/ml ^177^Lu-DOTA-chCE7 (300 μl) for 4 h. Cells were washed three times with PBS and incubated for 15 min with PBS/4% formaldehyde for fixation. Subsequently, cells were washed and incubated with 0.2% permeabilization buffer containing 0.03 M NaCl, 0.3 mM KH_2_PO_4_, 0.5 mM Na_2_HPO_4_ and 3% triton-X 100 diluted with PBS for 15 min at RT. Cells were blocked with PBS/1% BSA, 0.3% Tween-20 for 45 min and incubated over night with the primary anti-γH2A.X antibody (1/400, 4 °C, Cell Signaling Technology, Bioconcept, Allschwill, Switzerland) diluted in blocking buffer. Cells were incubated with a reaction mixture containing secondary fluorescein isothiocyanate (FITC)-conjugated antibody (1/300, Abcam, Cambridge, UK) and phalloidin (1/200, Cell Signaling Technology, Bioconcept, Allschwill, Switzerland) for 30 min at RT, washed three times and stored at 4 °C in the dark. Samples were imaged with a Leica SP5 confocal microscope (Leica Microsystems, Switzerland) using a 50 × 0.5NA dry objective. Fields were chosen at random locations. γH2A.X foci were calculated as a percentage of the total cells counted in each field.

### Apoptosis analysis via flow cytometry

Cells were treated with 300 nM MK1775 for 48 h and/or 5 MBq/ml ^177^Lu-DOTA-chCE7 (5 ml) for 8 h, detached, washed and subjected to apoptosis analysis via flow cytometry using an annexinV-FITC/propidium iodide (PI) double staining. Cells were incubated for 15 min with an annexinV-FITC/PI reaction mixture containing 10 mM Hepes/NaOH, pH 7.4; 140 mM NaCl; 5 mM CaCl_2_; as well as 20 μl of annexinV-FITC labelling reagent (Roche Diagnostics GmbH, Mannheim, Germany) and 20 μl PI solution (Flucka, Buchs, Switzerland). Labelled cells were transferred into a 96-well plate, analysed by flow cytometry and results were evaluated with FlowJo software (Tree Star, Ashland, OR, USA, version 10).

### In vivo studies

All animal experiments were approved by the cantonal committee on animal experiments and permitted by the responsible cantonal authorities (permission number 75666, Kanton Aargau). The studies were conducted in compliance with the Swiss laws on animal protection. For tumour growth, groups of 4–6 female CD-1^nu^ mice (Charles River, Sulzfeld, Germany, 5 weeks old, with an average weight of 22 g) were injected subcutaneously (s.c.) with 5 × 10^6^ IGROV1 cells (100 μl, in sterile PBS) into the right flank. One week before the radioimmunotherapy started, 200 μg of murine IgG2a (#M7769, Sigma-Aldrich, Buchs, Switzerland) was injected i.p. to minimize unspecific binding to murine Fcɣ and FcRn receptors. Nineteen days post tumour cell inoculation (mean tumour volume: 362 ± 150 mm^3^, mean body weight: 24.8 ± 1.6 g) mice were treated with a) 6 MBq (50% maximum tolerated activity (MTA) [20], 25 μg, 100 μl ^177^Lu-DOTA-chCE7, b) 6 MBq^177^Lu-DOTA-isotype control antibody (25 μg, 100 μl) or c) PBS into the tail vein. In some treatment groups MK1775 (50 mg/kg, CliniSciences, Nanterre, France) was given in DMSO in 0.5% methylcellulose (M0512; Sigma-Aldrich, Buchs, Switzerland) in a 1:14 suspension [37] by oral gavage on three consecutive days starting 48 h after RIT. The gavage needle was precoated with sucrose to reduce stress for the mice [38].

For the in vivo assessment of DNA damage, female CD-1^nu^ mice (*n* = 3; Charles River, Sulzfeld, Germany) were subcutaneously (s.c.) injected with 5 × 10^6^ SKOV3ip cells and 14 days later treated with 2 MBq of intravenously (i.v.) administered ^177^Lu-DOTA-chCE7 alone or in combination with 50 mg/kg MK1775 administered p.o. 48 h after RICs. PKI doses were administered daily for 3 consecutive days. Accordantly, controls received PBS. Six days post therapy start all animals were euthanized. Subcutaneous xenografts were measured, removed and fixed in 4% neutral-buffered formalin (Formafix, Hittnau, Switzerland) for 48 h. After fixation tissues were trimmed, dehydrated in graded alcohol and routinely paraffin wax embedded. Consecutive sections (3–5 μm thick) were prepared, mounted on glass slides and routinely stained with haematoxylin and eosin (HE) or subjected to immunohistochemical staining.

A rabbit polyclonal antibody against mouse phosphorylated γH2A.X histone antigen (antibody #2577, Cell Signaling Technology, Bioconcept, Allschwill, Switzerland) was used to detect endogenous levels of γH2A.X when phosphorylated at serine 139. Briefly, sections were deparaffinised in xylene rehydrated in decreasing concentrations of ethanol and subjected to antigen retrieval using 10 mM Tris-EDTA buffer (pH 9.0) for 15 min at 98 °C. This was followed by incubation for 15–18 h at 4 °C with the primary antisera (1/50 dilution in Dako antibody diluent, Dako-Agilent Technologies, Denmark). A detection kit containing the secondary antibody and diaminobenzidinetetrahydrochloride (DAB) as chromogen was subsequently applied according to the manufacturer’s protocols (Peroxidase/DAB+ Rabbit/Mouse Kit; DAKO-Agilent Technologies, Denmark), followed by light counterstain with hematoxylin.

An attempt was made to quantify the number of γH2A.X positive tumour cells. Slides were scanned using digital slide scanner NanoZoomer-XR C12000 (Hamamatsu, Japan) and images (10 images per sample, 40×) were taken using NDP.view2 viewing software (Hamamatsu). Fields were selected just beneath the capsule of the xenografts, avoiding areas exhibiting liquefactive necrosis. Immuno-stained cells for γH2A.X were calculated as a percentage of the total cells counted in each field.

### Statistical analysis

Statistical analysis of apoptosis, fluorescence microscopy, and histological data was performed using student’s t-test (two-tailed, unpaired) with Bonferroni-correction. Significance was determined with *p* < 0.0125. In vitro data was analysed via combination index calculations (CI = (*C*_A,*x*_/Ic_*x*,A_) + (*C*_B,*x*_/Ic_*x*,B_)). Thereby, concentrations required to produce a given effect are determined for drug A (Ic_*x*,A_) and drug B (Ic_*x*,B_). *C*_A*,x*_ and *C*_B,*x*_ are the concentrations of A and B contained in combination that provide the same effect. Synergy is determined for CI < 1, additivity for CI = 1 and antagonism for CI > 1 [39].

## Results

### Antibody radiolabelling and ligand substitution

In order to determine the DOTA-to-mAb (chCE7) ratio a mass spectroscopic analysis was performed. Results showed that an average of 7.6 chelators was coupled to one intact antibody molecule for RICs used in in vitro experiments. For RICs utilized in the in vivo study an average of 2.7–3.1 chelators was coupled. Specific activity ranged from 2000 to 2850 MBq/mg for RICs with 7.6 chelators and 240–560 MBq/mg for RICs with 2.7–3.1 chelators. Lindmo method [35] was used to prove the immunoreactive fraction of the radiolabelled mAbs (60–83%).

### Cytotoxicity of selected PKIs towards IGROV1 and SKOV3ip cells

**Fig. 1:**
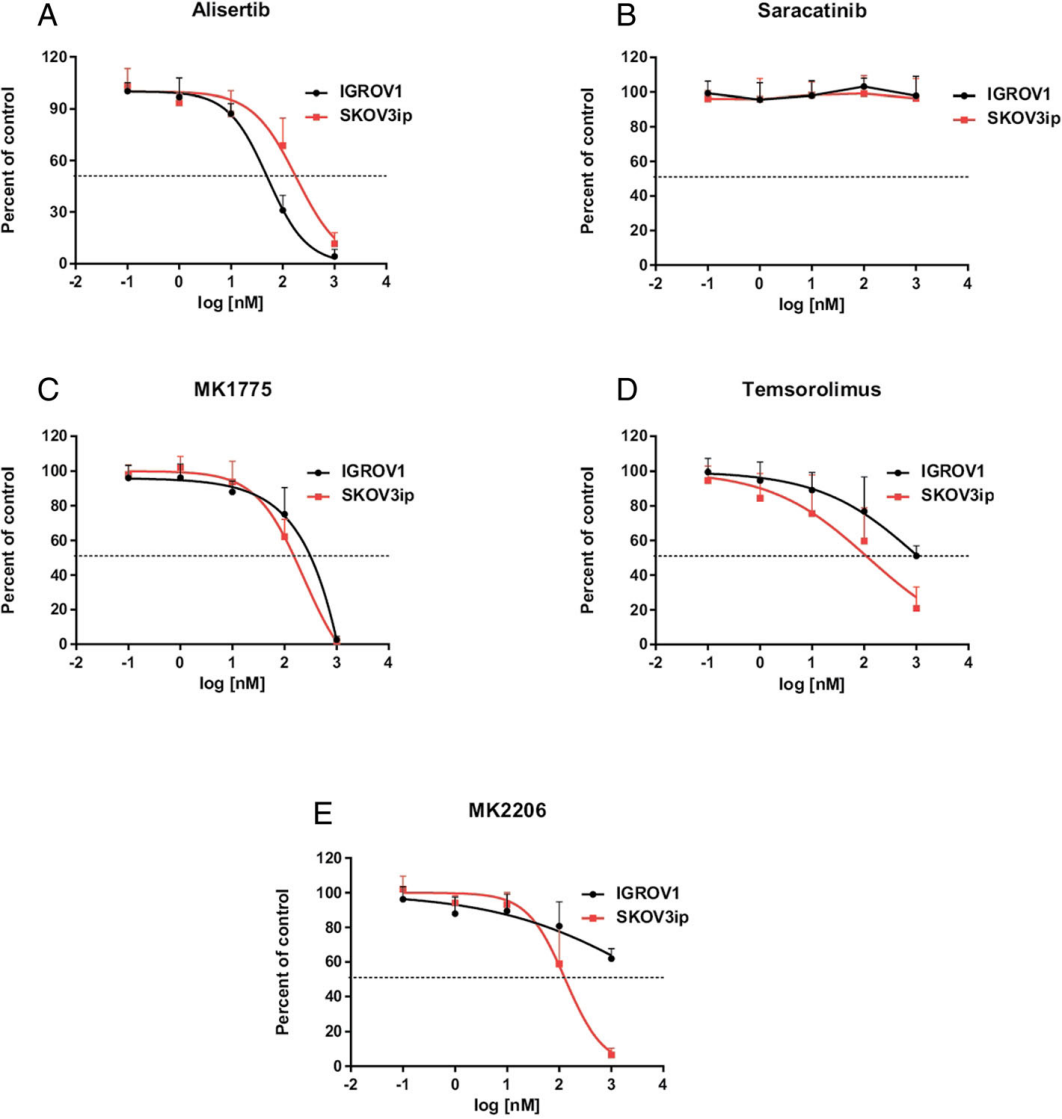
IC_50_-values for **a** Alisertib, **b** Saracatinib, **c** MK1775, **d** Temsirolimus and **e** MK2206. IC_50_-values were determined by colony-forming assays. IGROV1 and SKOV3ip cells were incubated for 48 h with accordant PKI concentrations ranging from 0.1–1000 nM

First we investigated the sensitivity of the IGROV1 and SKOV3ip OC cell lines towards the selected PKIs. Respective dose-response curves are shown in Fig. 1a-e and resulting IC_50_-values are summarised in Table 1. PKIs alisertib and MK1775 showed IC_50_-values in the medium nanomolar range for both OC cell lines. Comparable sensitivities of SKOV3ip cells were observed towards the PKIs temsirolimus and MK2206. In comparison, IC_50_-values for temsirolimus and MK2206 against IGROV1 cells could only be estimated within in the micromolar range, since highest applied PKI concentration of 1 μM was not sufficient enough to reach IC_50_. No cytotoxicity was detected for both cell lines based on the treatment with PKI saracatinib (Fig. 1b).

**Table 1:** IC_50_-values for PKIs against IGROV1 and SKOV3ip cells

	Alisertib	Saracatinib	MK1775	Temsirolimus	MK2206
IGROV1	50 ± 3 nM	n.a.	306 ± 4 nM	n.a.	n.a.
SKOV3ip	158 ± 3 nM	n.a.	133 ± 4 nM	120 ± 4 nM	131 ± 3 nM

*Abbreviation*: *n.a*. not available

### MK1775 sensitizes IGROV1 cells towards treatment with ^177^Lu-DOTA chCE7 in vitro

PKIs saracatinib, MK2206 and temsirolimus showed only limited cytotoxicities against both OC cell lines and were therefore not considered for further investigations. First in vitro combination experiments were performed using the PKIs alisertib and MK1775 combined with ^177^Lu-DOTA-chCE7. Both PKIs demonstrated their ability to radiosensitize IGROV1 cells by lowering the inhibitory concentration of ^177^Lu-labelled mAb chCE7 necessary to reduce colony-forming ability to 60% of an untreated control (IC_60_). However, MK1775 decreased the IC_60_-value of ^177^Lu-DOTA-chCE7 15-fold compared to a 6-fold decrease observed for combinations including PKI alisertib (Additional file 1: Figure S2). In this experiment the cells were incubated for only 4 h instead of 8 h with ^177^Lu-labelled chCE7. The colony-forming ability was reduced by a maximum of 60% in comparison to the untreated cells even with the highest amount of radioactivity.

Based on these findings MK1775 demonstrated the most promising radiosensitizing effect and was therefore further investigated. Following experiments included the examination of different treatment sequences for combined applications of MK1775 and ^177^Lu-DOTA-chCE7 in IGROV1 cells.

**Fig. 2:**
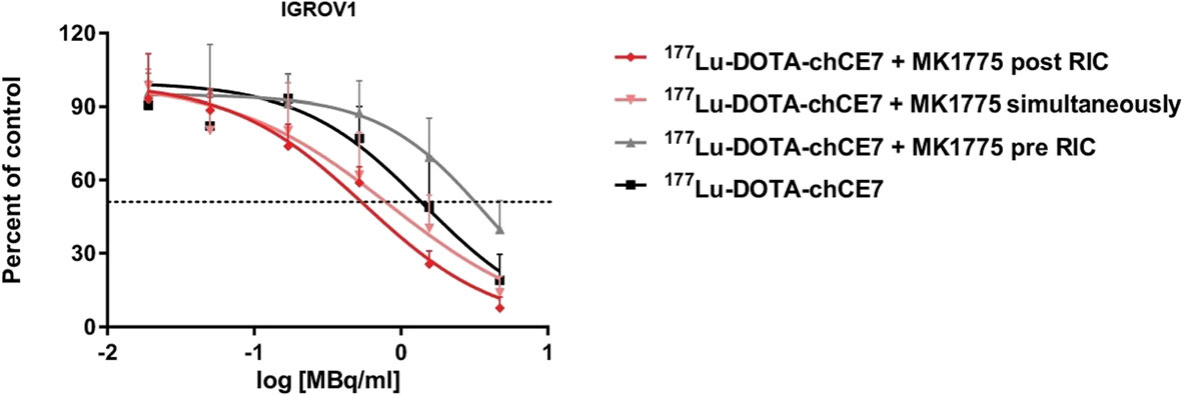
Radiosensitivity of IGROV1 cells. The radiosensitivity was determined by colony-forming assays after combined treatments with ^177^Lu-DOTA-chCE7 (range: from 0.01–5.0 MBq/ml, for 8 h) and MK1775 (applied simultaneously, 48 h post, or 48 h pre RIC with a concentration of half IC_50_)

The most prominent effects in IGROV1 were achieved when MK1775 (half IC_50_) was added simultaneously or post the radioimmunoconjugate (Fig. 2). IC_50_-value of ^177^Lu-DOTA-chCE7 was thereby lowered from 1.4 MBq/ml to 0.8 MBq/ml (simultaneous application), or down to 0.5 MBq/ml (MK1775 post RIC). MK1775 pre-treated cells showed no increased sensitivity towards the RIC but rather an increased IC_50_-value of 3.9 MBq/ml compared to ^177^Lu-DOTA-chCE7 alone. Combination index (CI) calculations as previously described by Zhao et al. [39] were used to determine synergy (Fig. 2, calculations are shown in Additional file 1: Table S1). The additional application of MK1775 (half IC_50_) post ^177^Lu-DOTA-chCE7 increased the cytotoxic effect of the RIC in a synergistic manner (CI < 1). The IC_50_ value for radioresistance towards ^177^Lu-DOTA-chCE7 was 4.7 MBq/ml for SKOV3ip cells (data not shown).

### The combination of MK1775 and RIC increases the amount of induced DNA-double strand breaks (DSBs) compared to monotreatments in IGROV1 cells

In order to elucidate the molecular mechanism behind the observed radiosensitizing effect of the Wee1 kinase inhibitor MK1775 on IGROV1 cells we investigated the phosphorylation status of cyclin-dependent-kinase 1 (alias CDC2), which is phosphorylated by protein kinase (PK) Wee1. Additionally, we correlated the phosphorylation of CDC2 (pCDC2) with the amount of induced DNA-DSBs by western blot analysis.

**Fig. 3:**
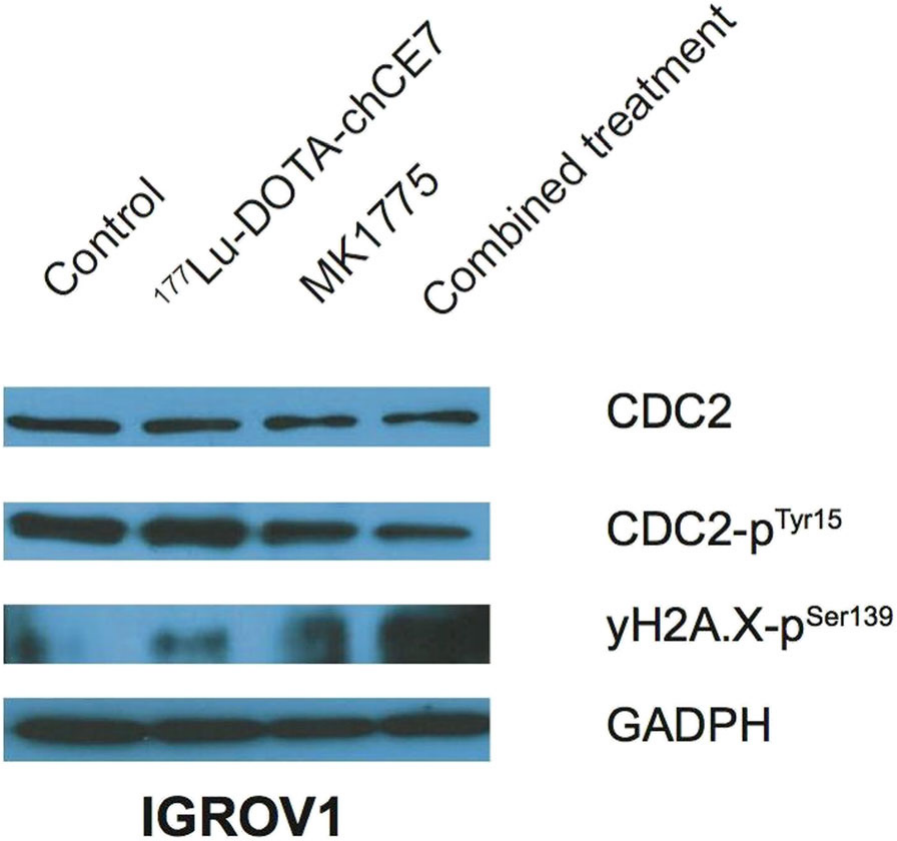
Western blot analysis of CDC2, phosphorylated CDC2 and phosphorylation of H2A.X in IGROV1 cell lysates. Cells were treated with either 5 MBq/ml ^177^Lu-DOTA-chCE7 for 8 h or 300 nM MK1775 for 48 h. For combination, cells were simultaneously incubated with both agents. After incubation (8 h) ^177^Lu-DOTA-chCE7 was removed and MK1775 further incubated until 48 h were reached. All cell lysates were taken directly post MK1775 treatment. Loading control: GADPH (glyceraldehyde 3-phosphate dehydrogenase)

It seems, that in IGROV1 cells expression levels of CDC2 were similar independent of a RIC and/or MK1775 application. Phosphorylation status of CDC2 (pCDC2) looks equal for the control and ^177^Lu-DOTA-chCE7. However, mono- or combined treatments containing MK1775 appears to reduce the levels of pCDC2 compared to controls or ^177^Lu-DOTA-chCE7 (Fig. 3).

Phosphorylation of histone H2A.X at Ser-139 (γH2A.X) was detected as an indicator for presence of DNA-DSBs. ^177^Lu-DOTA-chCE7 treated cells showed slightly higher amounts of γH2A.X compared to the control. The addition of MK1775 increased markedly the levels of γH2A.X compared to ^177^Lu-DOTA-chCE7 and the untreated cells. Combination of the RIC and PKI MK1775 showed the highest levels of γH2A.X, thus indicating the largest amount of induced DNA-DSBs compared to either treatment alone or the control (Fig. 3).

**Fig. 4:**
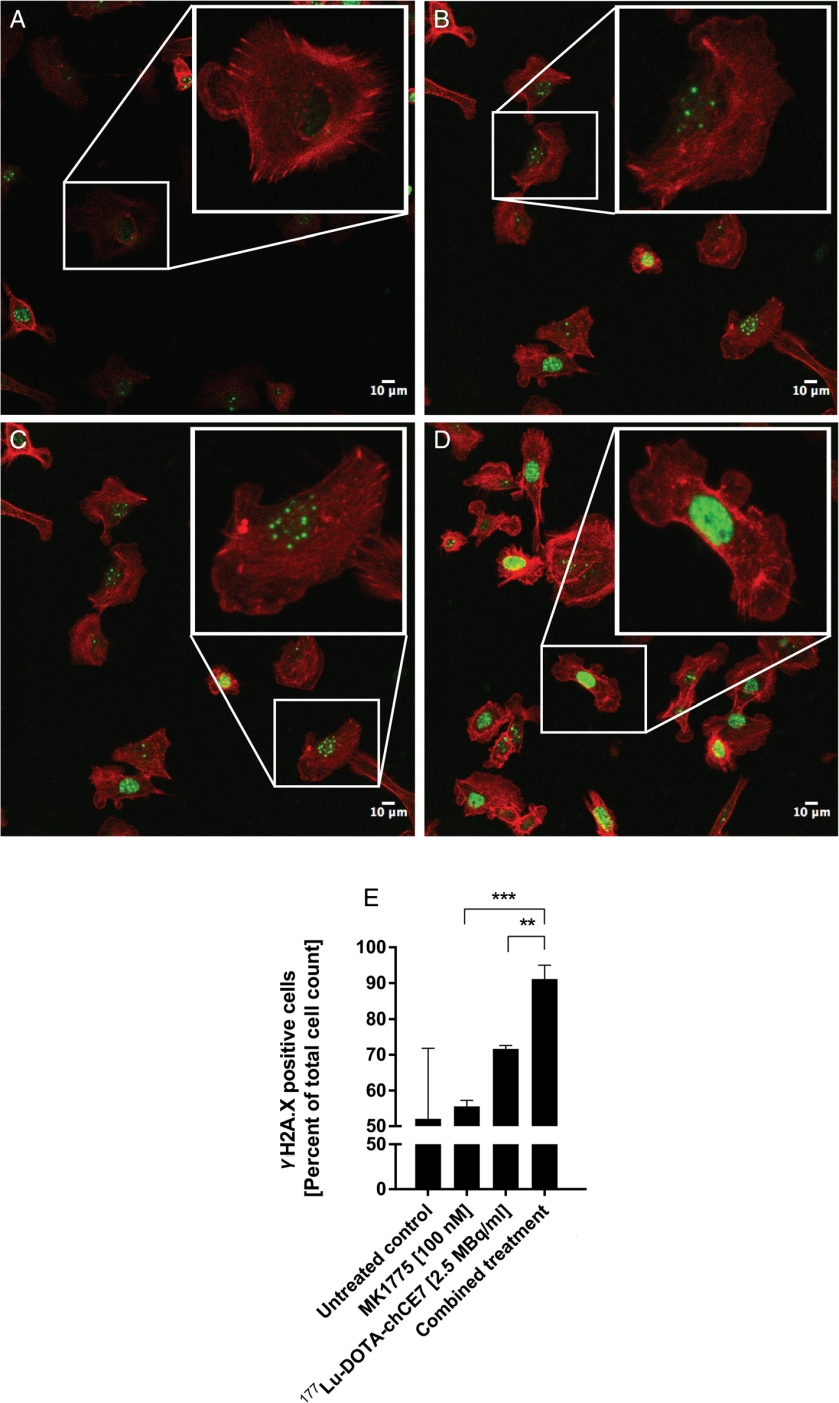
Fluorescence microscopy of γH2A.X. IGROV1 cells were incubated with 2.5 MBq ^177^Lu-DOTA-chCE7 for 4 h or with 100 nM MK1775 for 48 h. For combination, cells were simultaneously incubated with both agents. After 4 h incubation, ^177^Lu-DOTA-chCE7 was removed and MK1775 was further incubated until 48 h were reached. Cells were fixed and stained and γH2A.X foci were counted and cells grouped depending on (**a**) no observed (**b**) ≤ 5 (**c**) ≥ 6 or (**d**) non-distinguishable γH2A.X foci (intensively positive). **a-d** shows examples of IGROV1 cells with accordant numbers of γH2A.X foci. (**e**) summarizes all γH2A.X positive cells expressed in percent of total cell numbers counted

Fluorescence microscopy studies were conducted to quantify γH2A.X levels. The number of induced γH2A.X foci were counted and cells grouped depending on either no observed γH2A.X foci (Fig. 4a), count of foci per cell ≤5 (Fig. 4b), count of foci per cell ≥6 (Fig. 4c), or non-distinguishable γH2A.X foci (intensively positive, Fig. 4d). All counts are summarized in Additional file 1: Table S2. Figure 4e shows all γH2A.X positive cells expressed in percent of total cell numbers counted.

Non-treated IGROV1 cells were positively tested for γH2A.X foci (52 ± 19%), indicating that about half of IGROV1 cells show a basal level of existing of DNA-DSBs. After treatment with a low concentration of MK1775 (100 nM) IGROV1 showed comparable amounts of γH2A.X foci (55 ± 2%). In contrast, ^177^Lu-DOTA-chCE7 application resulted in an increased number of 72 ± 1%. Highest amounts of induced DNA-DSBs were observed for the combination demonstrating 91 ± 4% of IGROV1 cells being positive for γH2A.X foci. The total number of positive cells thereby significantly increased compared to either monotreatment (vs. MK1775: *p* < 0.0125; vs. ^177^Lu-DOTA-chCE7: p < 0.0125; Fig. 4e).

The number of cells showing ≤5 foci was similar for cells treated with ^177^Lu-labelled mAb or combination and amount of cells showing ≥6 foci was comparable all in groups (Additional file 1: Table S2). Interestingly, upon combination of both agents the number of intensively positive cells raised from 2 ± 1% (^177^Lu-DOTA-chCE7) and 5 ± 3% (MK1775) to 15 ± 7%. Based on these numbers, the significant increase in overall positive cells for the combination is mostly due to the higher amounts of intensively positive cells.

### The combination of MK1775 and RIC increase early-apoptosis compared to monotreatments in IGROV1

To investigate if induced DNA-DSBs result in apoptosis/necrosis, flow cytometry analysis was performed. Cells were either untreated (Additional file 1: Figure S3A), or incubated with MK1775 (Additional file 1: Figure S3B), ^177^Lu-DOTA-chCE7 (Additional file 1: Figure S3C), or both (Additional file 1: Figure S3D). AnnexinV-FITC/PI double staining was used to distinguish between early- and late-apoptosis/necrosis.

IGROV1 cells treated with ^177^Lu-DOTA-chCE7 demonstrated a doubling in early apoptotic cells compared to controls (^177^Lu-DOTA-chCE7: 8 ± 2%; control: 4 ± 1%). The incubation with 300 nM (IC_50_) MK1775 showed elevated levels of 29 ± 10% of cells being early apoptotic. Combination resulted in a significant higher number of early-apoptotic IGROV1 cells compared to ^177^Lu-DOTA-chCE7 alone (40 ± 4%; *p* < 0.0125) indicating that enhanced levels of induced DSBs (Fig. 3 and 4e) resulted in significantly increased early-apoptosis immediately after combined treatment, but not significantly compared to MK1775 monotreatment (*p* > 0.0125). The amount of late-apoptotic or necrotic cells did not vary significantly (Table 2).

**Table 2:** Percentage of early- and late-apoptotic/necrotic IGROV1 cells after treatment with MK1775 and ^177^Lu-DOTA-chCE7

IGROV1	Untreated control	MK1775 [300 nM]	177Lu-DOTA-chCE7 [5.0 MBq/ml]	Combined treatment
Early-apoptosis	4% ± 1	8% ± 2	29% ± 10	40% ± 4
Late-apoptosis/necrosis	9% ± 2	10% ± 2	13% ± 2	12% ± 1

The data were generated from three independent flow cytometry experiments

### Immunohistology reveals significant higher levels of DNA-DSBs in SKOV3ip xenografts administered with MK1775 as a single agent or in combination with ^177^Lu-DOTA-chCE7

**Fig. 5:**
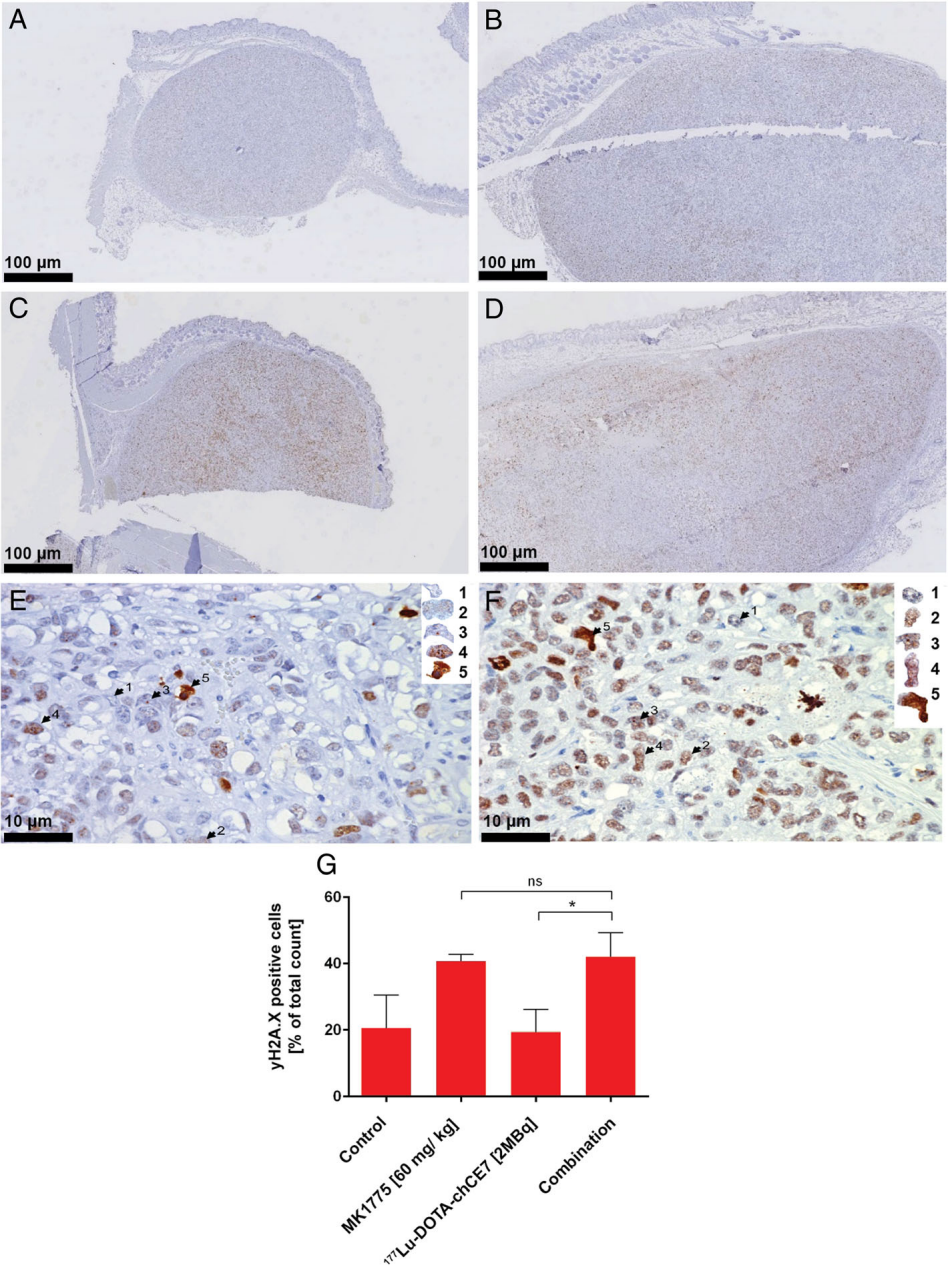
yH2A.X expression in SKOV3ip xenografts. Mice either received (**a**) PBS (**b**) 2 MBq ^177^Lu-DOTA-chCE7, (**c**) 50 mg/kg MK1775 p.o. for 3 consecutive days, or (**d**) combined treatment of ^177^Lu-DOTA-chCE7 with 50 mg/kg MK1775 p.o. for 3 consecutive days started 48 h post RIT. 10-fold higher magnifications are shown for (**e**) control tumours and (**f**) combination therapy tumour samples. γH2A.X foci were counted and cells grouped depending on 1) no observed, 2) diffuse, 3) ≤ 5, 4) ≥ 6, or 5) non-distinguishable γH2A.X foci (intensively positive). (**g**) summarizes counts of yH2A.X expressing cells in percent of total cell count

Two in vivo experiments were performed in order to assess whether the promising results from the in vitro work could be confirmed. First we analysed the immunohistochemical expression of the γH2A.X antigen in SKOV3ip xenografts after administration of MK1775 and ^177^Lu-DOTA-chCE7. A generalized increase in γH2A.X expression (brown staining) was evident in the xenografts exposed to MK1775 (used alone or in combination; Fig. 5c and d), when compared to those untreated (Fig. 5a) or exposed to ^177^Lu-DOTA-chCE7 monotreatment (Fig. 5b). A 10-fold higher magnification clearly demonstrates the difference in γH2A.X expression levels in controls (Fig. 5e) and combination therapy treated tumours (Fig. 5f). Observations were confirmed by γH2A.X foci counts (Fig. 5g), which were conducted following the criteria applied to in vitro immunofluorescence experiments. Control tumours and those received ^177^Lu-DOTA-chCE7 showed identical levels of γH2A.X foci (^177^Lu-DOTA-chCE7: 20 ± 7%; control: 21 ± 9%). In contrast, tumours treated with MK1775 or the combination therapy demonstrated similar, but higher amounts of γH2A.X foci (MK1775: 41 ± 2%; p < 0.0125; combination: 42 ± 8%, *p* > 0.0125) compared to the RIT or control.

### RIT with^177^Lu-DOTA-chCE7 and MK1775 reduces tumour growth in an IGROV-1 xenograft model

**Fig. 6:**
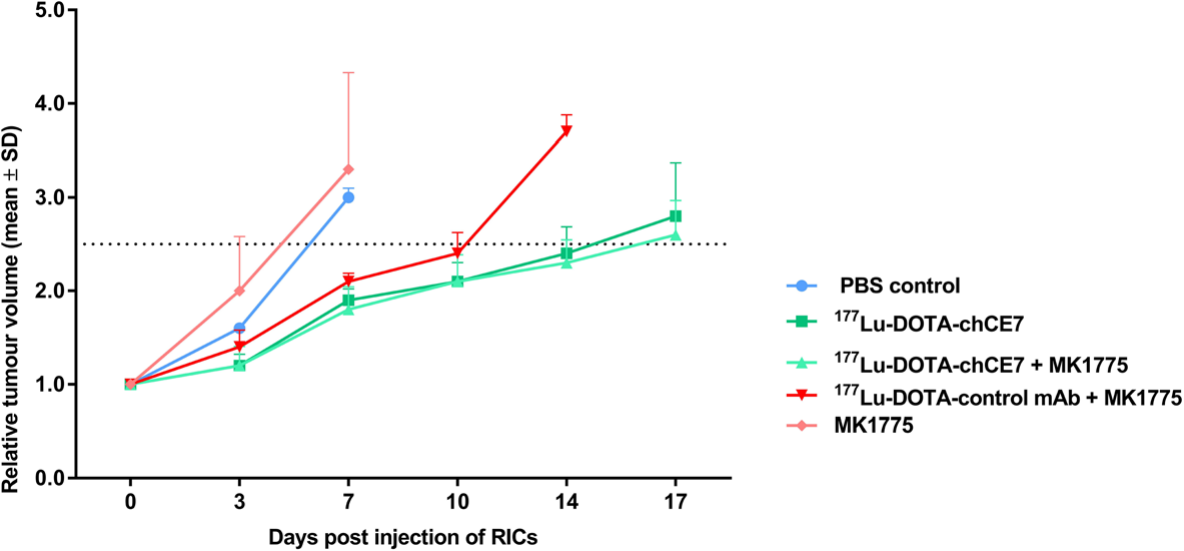
Therapeutic efficacy of anti-L1CAM lutetium-177 RIT in combination with MK1775 in nude mice bearing subcutaneous IGROV1 tumours. Tumour-bearing nude mice (*n* = 4–6) were treated with 6 MBq (50% MTA) ^177^Lu-DOTA-chCE7, 6 MBq ^177^Lu-DOTA isotype control mAb, combination of both radiolabelled antibodies with MK1775 (given on three consecutive days, starting 48 h after RIT), only MK1775 and only PBS. Tumour growth curves were stopped when the first tumour in a treatment group reached 1500 mm^3^. The dashed line represents a 2.5 increase in mean relative tumour volumes (± SD)

In a second in vivo study we analysed the tumour growth inhibition effect of a ^177^Lu-DOTA-chCE7 RIT in combination with MK1775. We have previously shown that the use of lutetium-177 labelled anti-L1CAM antibody chCE7 for RIT of ovarian cancer is suitable [17] and a combination with paclitaxel improved the outcome [19]. For this study we used an advanced tumour stage (mean tumour volume: 362 ± 150 mm^3^, maximal tumour size ≤700 mm^3^) to see whether RIT in combination with MK 1775 is able to curb a late stage of the disease. We observed a fast increase in tumour mass in the control mice and in the mice treated with MK1775 illustrated an unrestrained tumour growth (Fig. 6). In contrast, anti-L1CAM RIT alone or in combination with MK1775 markedly reduced tumour growth in comparison to the control group (PBS) and the MK1775 treated mice. The unspecific ^177^Lu-labelled control mAb in combination with MK1775 influenced the tumour growth to a moderate extent (Fig. 6). During therapy no signs of distress or lost of body weight of the mice were observed. Statistical calculations were omitted because of the modest sample size.

## Discussion

Initially, we investigated five clinically relevant PKIs (MK1775, alisertib, MK2206, temsirolimus and saracatinib; clinical phase I-II) towards their cytotoxicity against the two OC cell lines IGROV1 (p53wt) and SKOV3ip (p53del). PKIs MK1775 and alisertib demonstrated the highest toxicity against both cell lines and were further examined towards their ability to increase the efficacy of ^177^Lu-labelled mAb chCE7. Both PKIs led to a decreased IC_60_-value of the RIC demonstrating their ability to sensitize the p53wt celI line IGROV1 towards the ^177^Lu-labelled mAb. However, MK1775 was more efficient in combination with ^177^Lu-DOTA-chCE7 compared to the PKI alisertib. We therefore continued to investigate the influence of different treatment sequences of combined MK1775 and ^177^Lu-DOTA-chCE7 applications on IGROV1 cells. The influence of the p53 status for sensitization to radiation by MK1775 is contradictory in the literature and is discussed in detail by Geenen and Schellens [40].

MK1775-based sensitization of tumour cells towards DNA-damaging agents and EBRT, by abrogating the G2 cell cycle checkpoint and inhibiting DNA-repair, has been previously demonstrated in various cancer cell lines [26, 27, 41–43]. In our studies, the simultaneous application of ^177^Lu-DOTA-chCE7 and PKI MK1775, as well as a RIC pre-treatment resulted in 2–3-fold lower IC_50_-values compared to ^177^Lu-DOTA-chCE7 alone against IGROV1 cells. These results demonstrate the ability of MK1775 to sensitize IGROV1 cells towards RICs. Thus, our observations are in line with previous findings for combinations with EBRT [27, 43]. However, the addition of MK1775 prior the RIC showed no increased efficacy compared to ^177^Lu-DOTA-chCE7, pointing out the importance of treatment sequence investigations. Previously it has been shown that upon radiation-induced DNA damages the phosphorylation of CDC2 is maintained for 12 h in order to arrest the cell cycle in the G/2 M phase for DNA-repair [44]. We observed an increased radiosensitivity of IGROV1 cells when MK1775 was applied post or simultaneously with ^177^Lu-DOTA-chCE7, suggesting that both, the abolishment of already existing G2/M arrest and the inability to activate and maintain the G2/M arrest are forcing cells with damaged DNA into cell death.

We examined the induction of DNA-DSBs and apoptosis by mono- and combined treatments. Levels of histone H2A.X phosphorylation at Ser-139 (yH2A.X) was measured since it well correlates with the amount of existing DNA-DSBs [45]. Western blot analysis revealed slightly increased amounts of yH2A.X in ^177^Lu-DOTA-chCE7 treated IGROV1 cells compared to controls 40 h post RIC application. For EBRT it has been shown that radiation-induced DNA-damages could be repaired within 12-24 h post treatment [44]. However, since mAb chCE7 internalizes and deposits the therapeutic radionuclide ^177^Lu within the tumour cell, radiation exposure continues leading to increased amounts of present DBSs even after 40 h post ^177^Lu-DOTA-chCE7 removal.

Wee1-inhibiting MK1775 was shown to have multiple effects on tumour cells: First, replicative stress based on inactivated CDC2, enhanced initiation of DNA-replication and thus, shortage of nucleotides and lowered replication fork speed was observed [46]. Second, it was demonstrated that Wee1 also negatively regulates the Mus81-Eme1 endonuclease complex [47]. In turn, Wee1 inhibition most likely leads to increased Mus81-Eme1 endonuclease activity resulting in higher amounts of DNA-DSBs during DNA-replication. In line with these studies we showed enhanced levels of yH2A.X in IGROV1 cells after MK1775 applications compared to controls or ^177^Lu-DOTA-chCE7. Furthermore, the combined administration of MK1775 and ^177^Lu-DOTA-chCE7 showed higher levels of yH2A.X in IGROV1 cells compared to MK1775 or ^177^Lu-labelled mAb alone. This indicates that Wee1 inhibition prevented the repair of irradiation induced DNA-DSBs. Additional evaluation of induced yH2A.X foci via immunofluorescence confirmed previously obtained results showing significantly increased amounts of yH2A.X positive cells after combined application of MK1775 and ^177^Lu-DOTA-chCE7. The synergistic effect of MK1775 in combination with ^177^Lu-DOTA-chCE7 is therefore likely attributed to replicative stress and increased DNA damage induced by Wee1 inhibition.

Subsequent analysis of apoptosis/necrosis was performed to further examine if induced DNA-DSBs results in increased cell death. It has been shown in an osteosarcoma model that caspase activation upon combination of MK1775 and EBRT was significantly higher compared to only irradiated cells [43]. In our investigations, annexinV/PI analysis of IGROV1 cells revealed a significantly increased number of early-apoptotic cells for the combination of MK1775 with ^177^Lu-DOTA-chCE7 compared to ^177^Lu-DOTA-chCE7 treatment alone. This observation matches the previous findings from PosthumaDeBoer et al. [43]. Furthermore, our results demonstrate a correlation between the number of induced yH2A.X foci and increased early-apoptosis.

SKOV3ip showed a 3-fold higher IC_50_-value compared to IGROV1 cells for ^177^Lu-DOTA-chCE7. This observation supports the results of previous experiments with EBRT showing the highest radioresistance for SKOV3 cells compared to other OC cell lines [48]. Reasons for radioresistance have been recently discussed by Cojoc et al. [49]. Cancer cells exhibit multiple mechanisms to avoid DNA-damage upon radiation including (i) the regulation of the cell cycles status, (ii) an improved DNA-repair machinery and (iii) enhanced DNA protection against reactive oxygen species (ROS) [49]. The occurrence of these mechanisms certainly varies between cell lines and is most likely more distinct in SKOV3ip than in IGROV1 cells.

Immunohistology of SKOV3ip xenograft samples confirmed in vitro results showing high sensitivity against MK1775 and high radioresistance. The treatment with MK1775 for 4 days was enough, to induce elevated levels of yH2A.X in comparison to control and the treatment group with 2 MBq ^177^Lu-DOTA-chCE7. The combination therapy did not further increase the numbers of positive cells for yH2A.X. This suggests that MK1775 executes its toxicity in vivo, but additional application of 2 MBq ^177^Lu-DOTA-chCE7 did not produce further DNA-DSBs.

Increased therapeutic efficacy of EBRT in combination with MK1775 has been previously shown by Sarca et al. [50] in a glioblastoma model. Here we demonstrated that a combination of lutetium-177 RIT in combination with MK1775 in a late stage ovarian cancer model (IGROV1 xenograft) inhibited the tumour growth to a greater extent than the controls. The difference between RIT alone and the combination therapy was small and probably due to the modest group size. The influence of the lutetium-177 labelled unspecific antibody in combination with MK1775 on the tumour growth was moderate which is most likely due to a nonspecific accumulation of the mAb at the tumour site. Inflammations are sites where unspecific accumulation of radiolabelled antibodies occur [51]. The mice carried relative big tumours at therapy start and inflammation is often associated with tumour progression [52].

## Conclusion

To our knowledge this is the first time that RIT was combined with MK1775 and the results were reported. MK1775 radiosensitizes ovarian cancer cells and a combination with ^177^Lu-labelled anti-tumour antibody increased the efficacy of the treatments. Our results strongly support a further development of a combination of MK1775 with RIT.

## Additional file

Additional file 1: **Figure S1.** Flow cytometry analysis revealed that over 98% of IGROV1 and SKOV3ip cells expressed L1CAM on the cell surface. **Figure S2.** MK1775 showed most promising radiosensitizing effect in IGROV1 human OC cells. **Table S1.** Combination index (CI). Detailed information on the CI was shown. Data from Fig. 2 were used for the calculations. **Table S2.** Quantification of γH2A.X foci in IGROV1 cells treated with either 100 nM MK1775 (48 h), 2.5 MBq ^177^Lu-DOTA-chCE7 (4 h), or both. Detailed information about the γH2A.X foci (≤ 5/cell, ≥ 6/cell, Intensively positive cells) after treatments is shown in percent of total cell count. **Figure S3.** Apoptosis/necrosis analysis of IGROV1. AnnexinV-FITC/PI double staining was used to distinguish between early- and late-apoptosis/necrosis. Enhanced levels of induced DSBs resulted in significantly increased early-apoptosis immediately after combined treatment. (DOCX 410 kb)
