# Supplementary material for: Combination of lutetium-177 labelled anti-L1CAM antibody chCE7 with the clinically relevant protein kinase inhibitor MK1775: a novel combination against human ovarian carcinoma
Source: BMC Cancer. 2018 Sep 25;18:922. doi: 10.1186/s12885-018-4836-1 (PMC6156869; doi:10.1186/s12885-018-4836-1)
Supplement: Supplementary file 1 — Figure S1. Flow cytometry analysis revealed that over 98% of IGROV1 and SKOV3ip cells expressed L1CAM on the cell surface. Figure S2. MK1775 showed most promising radiosensitizing effect in IGROV1 human OC cells. Table S1. Combination index (CI). Detailed information on the CI was shown. Data from Fig. 2 were used for the calculations. Table S2. Quantification of γH2A.X foci in IGROV1 cells treated with either 100 nM MK1775 (48 h), 2.5 MBq 177Lu-DOTA-chCE7 (4 h), or both. Detailed information about the γH2A.X foci (≤ 5/cell, ≥ 6/cell, Intensively positive cells) after treatments is shown in percent of total cell count. Figure S3. Apoptosis/necrosis analysis of IGROV1. AnnexinV-FITC/PI double staining was used to distinguish between early- and late-apoptosis/necrosis. Enhanced levels of induced DSBs resulted in significantly increased early-apoptosis immediately after combined treatment. (DOCX 410 kb) [file 12885_2018_4836_MOESM1_ESM.docx]

**Additional file 1:**

**Combination of lutetium-177 labelled anti-L1CAM antibody chCE7 with the clinically relevant protein kinase inhibitor MK1775: a novel combination against human ovarian carcinoma**

Dennis Lindenblatt^1^, Nastassja Terraneo^1^, Giovanni Pelligrini^2^, Susan Cohrs^1^, Philipp René Spycher^1^, David Vukovic^3^, Martin Béhé^1^, Roger Schibli^1,4^, and Jürgen Grünberg^1^

^1^Center for Radiopharmaceutical Sciences ETH-PSI-USZ, Paul Scherrer Institute, Villigen PSI, Switzerland; ^2^Institut for Veterinary Pathology, University of Zurich, Switzerland; ^3^Department of Biochemistry University of Zurich; ^4^Department of Chemistry and Applied Biosciences, ETH Zürich, Switzerland

mail: dennis.lindenblatt@googlemail.com, giovanni.pellegrini@uzh.ch, [susan.cohrs@psi.ch](mailto:susan.cohrs@psi.ch), [philipp.spycher@psi.ch](mailto:philipp.spycher@psi.ch), dvukovic@student.ethz.ch, martin.behe@psi.ch, [roger.schibli@psi.ch](mailto:roger.schibli@psi.ch)

For correspondence or reprints contact: Jürgen Grünberg, Center for Radiopharmaceutical Sciences ETH-PSI-USZ, Paul Scherrer Institute, 5232 Villigen PSI, Switzerland; Phone +41-(0)56-3102848. Fax +41-(0)56-3102849, Email: [*juergen.gruenberg@psi.ch*](mailto:juergen.gruenberg@psi.ch)


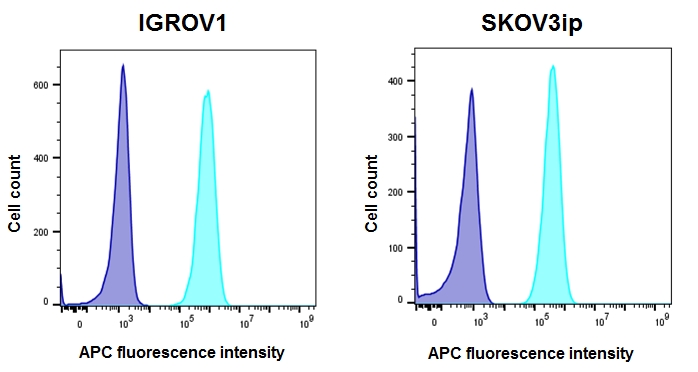


**Fig. S1** Flow cytometry analysis revealed that over 98% of IGROV1 and SKOV3ip cells expressed L1CAM on the cell surface. L1CAM expression is demonstrated by increased fluorescence intensity (turquoise peak). Untreated control cells (lilac) were used for background fluorescence. Primary antibody: chCE7, secondary antibody: goat F(ab')2 anti-human IgGFc (DyLight 650) (abcam, Cambridge, UK, # ab98593)


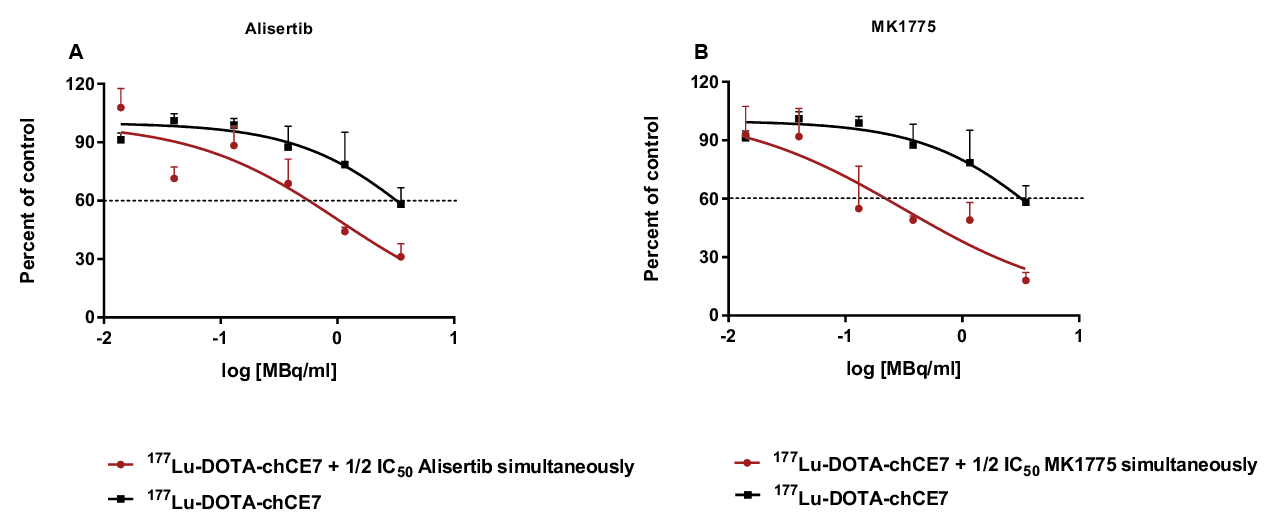


**Fig. S2** MK1775 showed most promising radiosensitizing effect in IGROV1 human OC cells. Combined treatment of ^177^Lu-DOTA-chCE7 (0.015-3.5 MBq/ml; 4h) A) Alisertib B) MK1775, both were applied simultaneously with ½ IC_50_.

IC_60_ of RIC:

3.1 MBq/ml for ^177^Lu-DOTA-chCE7

0.5 MBq/ml for ^177^Lu-DOTA-chCE7 + alisertib

0.2 MBq/ml for ^177^Lu-DOTA-chCE7 + MK1775

| **Table S1 Combination index (CI)**  **Data from Fig. 2 were used for the calculations** | | |
| --- | --- | --- |
| **Combination** | **^177^Lu-DOTA-chCE7 concentration to achive IC_50_ when ½ IC_50_ MK1775 [150 nM] was added** | **Combination Index** |
| ^177^Lu-DOTA-chCE7 + MK1775 post RIC | 0,5 MBq/mL | 0.86 |
| ^177^Lu-DOTA-chCE7 + MK1775 Simultaneously | 0,8 MBq/mL | 1,07 |
| ^177^Lu-DOTA-chCE7 + MK1775 pre RIC | 3,9 MBq/mL | 3,29 |

Combination index calculations (CI = (CA,x/Icx,A) + (CB,x/Icx,B)). Concentrations required to produce a given effect are determined for drug A (Icx,A) and drug B (Icx,B). CA,x and CB,x are the concentrations of A and B contained in combination that provide the same effect. Synergy is determined for CI < 1, additivity for CI = 1 and antagonism for CI > 1. IC_50_ for monotreatmens: MK1775: ≈ 300 nM, ^177^Lu-DOTA-chCE7: 1.4 MBq/mL.

| Table S2 Quantification of γH2A.X foci in IGROV1 cells treated with either 100 nM MK1775 (48h), 2.5 MBq 177Lu-DOTA-chCE7 (4h), or both. Results shown in percent of total cell count. | | | | |
| --- | --- | --- | --- | --- |
| IGROV1 | Untreated cells | MK1775 (100 nM) | 177Lu-DOTA-chCE7  (2.5 MBq/ml) | Combined treatment |
| Negative cells | 46 ± 16 | 44 ± 1 | 28 ± 1 | 9 ± 4 |
| Count of foci per cell ≤ 5 | 30 ± 8 | 28 ± 3 | 53 ± 2 | 51 ± 6 |
| Count of foci per cell ≥ 6 | 21 ± 12 | 22 ± 4 | 17 ± 2 | 24 ± 2 |
| Intensively positive cells | 1 ± 2 | 5 ± 3 | 2 ± 1 | 15 ± 7 |


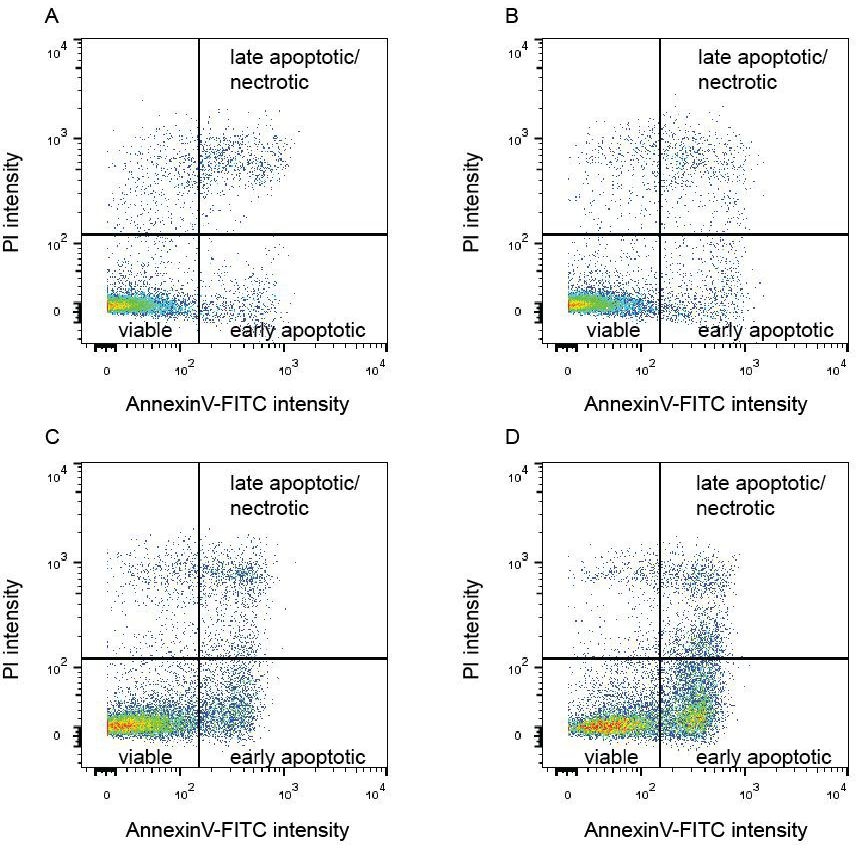


**Fig. S3** Apoptosis/necrosis analysis of IGROV1. Cells were double-stained with an annexinV-FITC/PI solution to distinguish between early- or late-apoptosis/necrosis after A) no treatment, B) MK1775 (300 nM, 48h), C) ^177^Lu-DOTA-chCE7 (5 MBq/ml, 8h), or D) combined treatment (after 8h incubation, ^177^Lu-DOTA-chCE7 was removed and MK1775 was further incubated until 48h were reached).
